# A Lightweight Neural Network Based on Memory and Transition Probability for Accurate Real-Time Sleep Stage Classification

**DOI:** 10.3390/brainsci15080789

**Published:** 2025-07-25

**Authors:** Dhanushka Wijesinghe, Ivan T. Lima

**Affiliations:** Department of Electrical and Computer Engineering, North Dakota State University, Fargo, ND 58105, USA; dhanushka.wijesinghe@ndsu.edu

**Keywords:** autocovariance, EEG, feedforward neural network, memory-augmented neural network, portable sleep monitoring, real-time classification

## Abstract

**Background/Objectives**: This study shows a lightweight hybrid framework based on a feedforward neural network using a single frontopolar electroencephalography channel, which is a practical configuration for wearable systems combining memory and a sleep stage transition probability matrix. **Methods**: Motivated by autocorrelation analysis, revealing strong temporal dependencies across sleep stages, we incorporate prior epoch information as additional features. To capture temporal context without requiring long input sequences, we introduce a transition-aware feature derived from the softmax output of the previous epoch, weighted by a learned stage transition matrix. The model combines predictions from memory-based and no-memory networks using a confidence-driven fallback strategy. **Results**: The proposed model achieves up to 85.4% accuracy and 0.79 Cohen’s kappa, despite using only a single 30 s epoch per prediction. Compared to other models that use a single frontopolar channel, our method outperforms convolutional neural networks, recurrent neural networks, and decision tree approaches. Additionally, confidence-based rejection of low-certainty predictions enhances reliability, since most of the epochs with low confidence in the sleep stage classification contain transitions between sleep stages. **Conclusions**: These results demonstrate that the proposed method balances performance, interpretability, and computational efficiency, making it well-suited for real-time clinical and wearable sleep staging applications using battery-powered computing devices.

## 1. Introduction

Sleep plays a fundamental role in maintaining both physical and mental well-being. It supports critical functions such as memory consolidation, cellular repair, and overall brain development [1,2,3]. While there is no universally accepted definition of good sleep, some researchers describe it as the structured progression through distinct sleep stages, regulated by neuro-chemical processes that contribute to overall health [4]. Sufficient sleep is essential for cognitive function, emotional stability, and general quality of life. The assessment of sleep patterns is particularly valuable for diagnosing and managing sleep-related disorders, including sleep apnea, insomnia, narcolepsy, schizophrenia, and depression [3,5,6]. In the United States alone, an estimated 50 to 70 million individuals suffer from chronic sleep disturbances, with approximately one-third of adults failing to meet recommended sleep durations [7]. These statistics underscore the necessity for accurate and objective methods to evaluate sleep quality and structure. Sleep staging, which involves identifying different sleep phases, plays a crucial role in both clinical diagnosis and therapeutic interventions for sleep disorders.

Sleep is categorized into distinct stages, broadly classified into non-rapid eye movement (NREM) and rapid eye movement (REM) sleep. NREM sleep consists of four stages: N1, N2, N3, and N4, each characterized by different brainwave activity, muscle tone, and physiological functions [3]. N1 is the lightest stage, marking the transition from wakefulness to sleep, while N2 involves a further decrease in responsiveness and the presence of sleep spindles and K-complexes, which are important for memory processing. N3 and N4, often referred to as deep sleep or slow-wave sleep, are critical for physical restoration, immune function, and overall recovery. REM sleep, on the other hand, is associated with vivid dreaming, memory consolidation, and cognitive processing [8]. Throughout the night, individuals cycle through these stages approximately every 90 min, with REM periods becoming longer in later cycles [3]. The proper alternation of these stages is essential for overall sleep quality, and disruptions in this cycle are often linked to sleep disorders and cognitive impairments [9].

The gold standard for sleep analysis is polysomnography (PSG), a comprehensive diagnostic tool that records multiple physiological signals, including electroencephalography (EEG) for brain activity, electromyography (EMG) for muscle movement, electrooculography (EOG) for eye movement, and additional parameters such as heart rate, oxygen levels, and respiratory effort [10]. PSG is typically conducted in sleep laboratories under controlled conditions, providing high accuracy in sleep staging and disorder diagnosis. However, it is time-consuming, expensive, and requires expert supervision, making it less accessible for large-scale or long-term sleep monitoring [11]. Sleep staging from PSG data is traditionally performed using manual scoring, where trained sleep experts classify each time segment (denoted epoch) of sleep into predefined stages based on visual inspection of EEG, EOG, and EMG patterns. The two primary techniques used for manual scoring are Rechtschaffen and Kales (R&K) criteria and the American Academy of Sleep Medicine (AASM) guidelines [12,13]. The R&K system, developed in 1968, was the first standardized method for six-class sleep classification, while the AASM guidelines, introduced later, refined the criteria by reducing the number of NREM stages by combining N3 and N4 stages and incorporating updated scoring rules. Although manual scoring is highly accurate, it is labor-intensive, prone to inter-scorer variability, and impractical for real-time or large-scale sleep studies [14]. These limitations have driven the development of automated sleep classification methods using machine learning and deep learning techniques.

With the rapid advancement of EEG-based wearable devices, there is an increasing demand for automated sleep scoring methods that can operate efficiently on resource-constrained hardware using a smaller number of electrodes, preferably single-channel [15]. Wearable EEG devices provide a convenient and non-intrusive way to monitor sleep outside of clinical settings, enabling long-term sleep tracking and personalized health insights. However, due to their limited computational power, storage, and battery life, traditional high-complexity models are not always feasible for real-time analysis [16]. Convolutional neural networks (CNNs) and recurrent neural networks (RNNs) are among the most widely used deep learning models for automated sleep staging. CNNs excel at extracting spatial patterns from EEG signals, while RNNs are effective in capturing temporal dependencies. Despite having higher accuracy than the other less computationally intensive models, CNNs and RNNs require substantial computational resources, memory, and processing time, making them less suitable for deployment on lightweight wearable devices. To overcome these challenges, there is a need for a reliable, efficient, and accurate sleep classification method that minimizes computational complexity while maintaining high performance.

**Figure 1:**
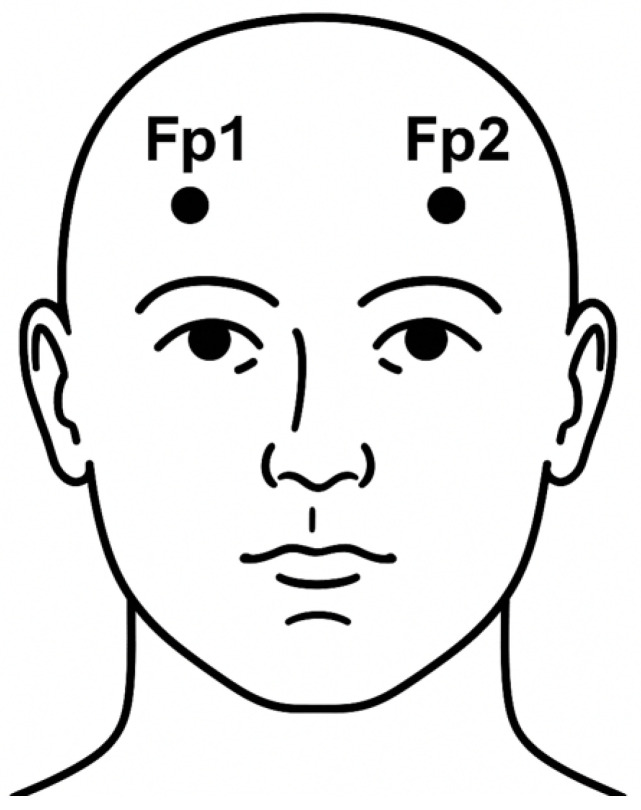
Frontal view of the human head illustrating the positions of the Fp1 and Fp2 electrode placements based on the international 10–20 system.

Even though a wide range of studies have demonstrated high classification performance in automatic sleep staging using machine learning, these results have often been achieved using EEG channels that are considered optimal for sleep analysis, such as F4–EOG, C4–EOG, or Fpz–Cz. These channels provide rich information for distinguishing between sleep stages but typically require precise electrode placement and technical expertise, which can be impractical in home-based or non-clinical environments. For practical, large-scale deployment—particularly in ambulatory or consumer-grade systems, ease of setup and user comfort are critical. In this context, the Fp1–Fp2 channel, located on the forehead, offers a highly accessible alternative due to its simplicity in placement and minimal preparation requirements. Figure 1 shows the location of the Fp1–Fp2 electrode placements, which represent a convenient and comfortable configuration for portable sleep monitoring. However, this convenience comes at the cost of reduced signal fidelity and increased susceptibility to noise and artifacts, posing significant challenges for robust sleep stage classification. Moreover, there remains a notable gap in the literature concerning the use of Fp1–Fp2 for sleep staging, particularly in the context of machine learning approaches.

In this study, we propose a novel approach to improve sleep stage classification using the Fp1–Fp2 channel by incorporating temporal context through a lightweight yet effective method. Our central hypothesis is that sleep stages exhibit finite temporal dependencies—meaning the stage of a given epoch is probabilistically influenced by the preceding stage. To validate this hypothesis, we analyzed two publicly available datasets and evaluated the autocovariance profiles of individual sleep stages, confirming that their temporal correlations extend well beyond a single 30 s epoch. Building on this insight, we introduce a transition-aware feature vector derived from the softmax output of the previous epoch, weighted by a transition matrix that captures learned or empirical stage-to-stage transition probabilities. This transition vector is incorporated into our feedforward neural network (FNN) alongside conventional time- and frequency-domain EEG features. Unlike sequential deep learning models such as CNNs and RNNs, which require entire sequences and are computationally intensive, our model selectively captures temporal context through this compact, interpretable representation. This results in improved classification performance and inter-stage continuity, while preserving low computational overhead—making the method highly suitable for real-time sleep staging in portable and wearable EEG systems.

## 2. Related Work

Automatic sleep scoring has been an active area of research for several decades, with significant advancements driven by the development of machine learning algorithms. The evolution of sleep staging techniques has seen a transition from traditional PSG setups with multiple physiological signals to more streamlined models utilizing fewer channels, with some approaches reducing the input to a single EEG channel. Sharma et al. utilized two EEG channels (C3-A2 and C4-A1), one EMG channel, and two EOG channels, achieving classification accuracies of 84.3% and 86.3% on the SHHS-1 and SHHS-2 databases, respectively [17]. Similarly, Tzimourta et al. employed six EEG channels from the ISRUC-Sleep dataset, obtaining a maximum accuracy of 75.29% [18]. Kong et al. used two EEG channels (Fpz-Cz and Pz-Oz), one EOG channel, one chin EMG channel, and event markers from the Sleep-EDF-20 and Sleep-EDF-78 datasets, reporting average accuracies of 82.7% and 80.0%, respectively [19]. While these studies demonstrated the effectiveness of multi-channel PSG-based sleep staging, such setups can be cumbersome and impractical for long-term, home-based monitoring. Although wearable sleep monitoring devices with reduced channel configurations exist, they can still cause discomfort during sleep [20,21]. Recent research has demonstrated that even a single-channel EEG signal is sufficient for sleep staging, following the five-stage classification defined by the AASM or the six-stage system by R&K [22,23,24,25,26,27]. Given our objective of developing a low-complexity, computationally efficient neural network model suitable for EEG-based wearable devices, this section focuses on key advancements in five- or six-stage sleep stage classification using single-channel EEG.

Traditional machine learning techniques, such as Random Forest, Support Vector Machine, K-Nearest Neighbors, Linear Discriminant Analysis, and Naïve Bayes classifiers, have been extensively used for single-channel or multi-channel EEG-based sleep stage classification, leading to notable improvements in accuracy and model agreement [22,28,29,30]. However, due to the highly nonlinear nature of brain activity, neural networks have gained significant attention in sleep staging research [31]. While FNNs represent the simplest form of neural networks, utilizing a straightforward hidden layer architecture with relatively low computational cost, most research has focused on more complex architectures such as CNNs and RNNs [19,23,24,26]. This preference is driven by the fact that sleep stages exhibit temporal dependencies, which CNNs and RNNs can leverage to enhance classification performance.

CNNs are a type of feedforward neural network that incorporate convolutional operations, making them highly effective for feature extraction [32]. While CNNs are primarily designed for image processing, their one-dimensional (1D) variants have been successfully applied to sleep staging [32]. Tsinalis et al. utilized class-balanced random sampling within the stochastic gradient descent optimization process to train a CNN model on single-channel (Fpz-Cz) EEG data from the PhysioNet EEG dataset [26]. Their approach achieved a high mean accuracy across individual sleep stages (82%, range 80–84%), and an overall accuracy of 74% (range 71–76%) across all subjects. Supratak et al. developed DeepSleepNet, which combines CNNs for time-invariant feature extraction with bidirectional long short-term memory networks to model sleep stage transitions [23]. Their model, tested on the MASS and Sleep-EDF datasets, achieved overall accuracies of 86.2% and 82.0%, respectively. Similarly, Liao et al. introduced LightSleepNet, a single-channel (Fpz-Cz) EEG-based CNN model designed for high accuracy and computational efficiency [33]. The authors demonstrated that LightSleepNet could be deployed on mobile platforms with limited hardware resources while achieving state-of-the-art performance, with an overall accuracy of 83.8% on the Sleep-EDF dataset.

Compared to CNN-based approaches, relatively fewer studies have explored the use of RNNs for sleep staging. Phan et al. proposed a feature learning approach using deep bidirectional RNNs with an attention mechanism for single-channel (Fpz-Cz) automatic sleep stage classification [34]. They tested their model on the Sleep-EDF dataset and achieved a maximum of 82.5% overall accuracy. Xueyan et al. proposed a hybrid architecture that integrates the bidirectional long short-term memory RNN network with a CNN, termed Bi-LSTM-CNN, to perform automatic sleep classification using multi-channel sleep data. This Bi-LSTM-CNN model has shown an accuracy of 89.4% on 39 samples of the Sleep-EDF dataset. Phan et al. also proposed the SeqSleepNet algorithm, which employs an end-to-end hierarchical RNN architecture [35]. This model processes multiple consecutive epochs simultaneously to predict sleep stage labels in a single output. By leveraging data from three channels—EEG, EMG, and EOG—it achieves an overall accuracy of 87.1% on the MASS SS3 dataset.

One of the major limitations of CNN- and RNN-based sleep staging models is their reliance on input data of a large number of prior epochs for accurate classification of the current epoch. For instance, DeepSleepNet requires 25 epochs (equivalent to 12.5 min) of raw EEG data to be processed together in order to generate sleep stage labels [23,36]. This dependency arises primarily due to the use of Bi-LSTM, which relies on long temporal sequences to enhance accuracy. Similarly, all other CNN- or RNN-based models, such as the CNN-based approaches by [26,37] as well as SeqSleepNet [35], also require long temporal sequences for inference, using 4, 5, and 10 consecutive raw EEG epochs (each 30 s long), respectively. The computational demands of these models, driven by the need to process large amounts of sequential data, make them unsuitable for real-time applications on wearable devices, which have limited processing capabilities and operate on battery power [15]. This constraint highlights the need for more efficient, lightweight sleep staging models that can function reliably with minimal computational resources.

To overcome the limitations of existing CNN- and RNN-based sleep staging models, we propose a computationally efficient approach by incorporating output feedback into a basic neural network architecture. Inspired by the long autocorrelation time of the sleep stage classifications, which are Bernoulli processes, our method introduces prior epoch classification (memory) as an additional feature for predicting the current epoch’s sleep stage. Unlike CNN and RNN models, which require a large sequence of prior input features for classification, our approach leverages only the output of previous epochs along with the time-domain and frequency-domain features of the current epoch. This significantly reduces computational complexity while maintaining high classification accuracy. Furthermore, we systematically investigated the optimal number of prior epochs to include as feedback in order to maximize model performance. To the best of our knowledge, this is the first study to explore the integration of output feedforward from a previous stage in EEG-based sleep staging, making it a promising solution for real-time, wearable sleep monitoring applications.

There are a few studies in which researchers exploited transitional rules of sleep to improve the sleep stage classification accuracy. While those approaches differ from our proposed method, they highlight the effectiveness of incorporating prior epoch memory to refine sleep classification. For example, Huang et al. introduced a sleep staging model based on single-channel EEG data, utilizing two convolution kernels of different scales to automatically extract features [38]. The model initially predicts sleep stages based on these learned features, and a Hidden Markov Model is subsequently applied to enforce sleep transition rules, refining the final classification. When tested on the Sleep-EDF dataset, this approach achieved an accuracy of 84.6% on the Fpz-Cz channel. Similarly, Bufang et al. proposed a contextual refinement algorithm based on conditional random fields to improve sleep staging predictions [39]. This algorithm functions as a postprocessing step to correct unreasonable sleep stage transitions in hypnograms generated by pre-classifiers. When paired with CNN-based classifiers, their method improved overall accuracy by 2.5–5.5% across multiple public datasets. These data further support our hypothesis that integrating previous epoch classifications can improve model performance without adding significant computational complexity, making it particularly well-suited for real-time, low-power applications.

A comparative summary of recent multi-channel and single-channel EEG-based sleep staging models is presented in Table 1. This table outlines each study’s model type, EEG setup, dataset, performance, and suitability for real-time applications. It highlights the gap that our method aims to address: achieve high performance with minimal computational cost using a simple, memory-augmented feedforward network.

## 3. Materials and Methods

**Figure 2:**
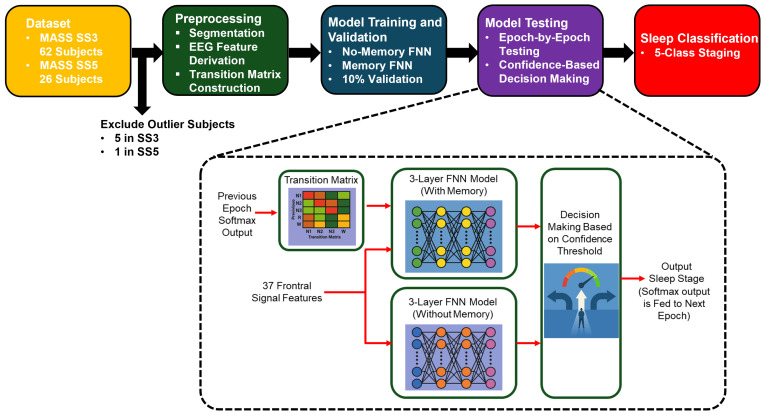
Overview of the proposed sleep staging pipeline. The process begins with EEG recordings from the MASS SS3 and SS5 datasets, followed by feature extraction and transition matrix construction. Two feedforward neural networks are trained: a no-memory model using EEG features alone and a memory-augmented model that incorporates temporal context via a transition matrix and prior softmax outputs. During testing, predictions are filtered using a confidence threshold, and final classification is performed using the more confident model or rejected if both are uncertain.

Figure 2 presents a high-level overview of our proposed sleep staging pipeline. It outlines the major stages of the workflow, including data preprocessing, model training (with and without memory), and confidence-based decision-making. This visual summary provides context for the detailed methodology described in the following subsections.

### 3.1. Dataset

In this study, we evaluated our sleep staging framework using the Montreal Archive of Sleep Studies (MASS), specifically the datasets SS3 and SS5 [40]. Both datasets include comprehensive polysomnographic recordings comprising EEG, EOG, EMG, and ECG channels. The SS3 subset consists of overnight PSG recordings from 62 healthy adult participants (29 males, 33 females), with a mean age of 42.5 ± 18.9 years (range: 20–69 years). The recordings adhere to the AASM guidelines and are annotated in 30 s epochs. Each subject’s PSG includes 20 EEG channels referenced to a linked-ear reference (LER, 10 kΩ impedance), 2 EOG channels, 3 referential EMG channels, and 1 ECG channel. The SS5 subset includes recordings from 26 healthy subjects (13 males, 13 females), with a mean age of 25.0 ± 7.4 years (range: 20–59 years). These recordings follow the R&K scoring system and are segmented into 20 s epochs. The PSG setup is similar to SS3, with 20 EEG electrodes (LER referenced), 2 EOG channels, 3 EMG channels, and 1 ECG channel.

Inclusion criteria for both SS3 and SS5 datasets were as follows:

- Healthy adult subjects without known neurological or psychiatric disorders;
- Availability of full-night PSG recordings with corresponding expert sleep stage annotations;
- No significant data loss or recording artifacts in the EEG channels used.

Addtional exclusion criteria:

- Subjects identified as statistical outliers based on EEG feature distributions (greater than 1.5 times the interquartile range from the global median);
- Subjects exhibiting abnormal sleep dynamics, such as excessive stage transitions (>40 transitions per hour), which may suggest labeling or recording errors.

Based on these criteria, five participants were excluded from SS3—four due to extreme deviations in feature distributions and one due to an anomalously high transition rate. One subject was excluded from SS5 due to outlier EEG features.

The SS3 dataset includes five-class expert annotations (wake, N1, N2, N3, and REM), whereas the SS5 dataset initially uses a six-class scheme. For consistency with SS3 and to align with standard practice, stages N3 and N4 in SS5 were merged into a single N3 stage, resulting in a unified five-class labeling format.

Given the focus on practical and user-friendly sleep monitoring, we utilized the Fp1–Fp2 EEG channel, which is particularly suitable for wearable systems due to its ease of placement. The Fp1–Fp2 signals from the datasets were obtained by computing the voltage difference between the Fp1–LER and Fp2–LER channels, where LER refers to the linked-ear reference.

### 3.2. Autocovariance Calculation

The numerical estimate of the autocovariance function of the indicator function of each sleep stage $I_{t}$ for all the participants, shown in (1), was computed using MATLAB R2024a,

(1) $$\gamma \left(\tau \right)=\frac{1}{n}\sum_{t=t_{1}}^{t_{n}-\left|\tau \right|}\left(I_{t+|\tau |}-\bar{I}\right)\left(I_{t}-\bar{I}\right),$$

where $\bar{I}$ is the sample mean of the sleep stage indicator function, τ is the time shift, and *n* is the number of time samples in the range. The estimate of the autocovariance function of $I_{t}$ is assumed to be ergodic to enable γ(τ) to be estimated using samples at different times.

To simplify the calculation of the autorrelation time of each sleep stage, the autocovariance function γ(τ) of that sleep stage is normalized by its variance, γ(0). The resulting normalized autocovariance function ρ(τ) is

(2) $$\rho \left(\tau \right)=\frac{\gamma (\tau )}{\gamma (0)}.$$

The estimated normalized covariance functions for each sleep stage indicator function were obtained by averaging all the autocovariance functions of all the participants. The autocorrelation time of each sleep stage was calculated using the discrete autocorrelation time equation that is given by

(3) $$t_{c}=\sum_{\tau =0}^{\tau_{\max}}\rho \left(\tau \right)\Delta t,$$

where Δ*t* is the discrete time interval and $\tau_{\max}$ is the maximum lag considered. Since negative correlation indicates an inverse relationship between time-lagged values of the indicator function, which does not reflect persistence of the same sleep stage over time, $\tau_{\max}$ was selected as the maximum time shift before the first zero-crossing of the autocovariance function. This approach is common in estimating integral correlation times, as only positive autocorrelations contribute to sustained memory or persistence in the signal [41]. Including negative values could bias the estimate downward and obscure the effective duration of temporal dependencies relevant for modeling.

### 3.3. Feature Extraction for Machine Learning Models

All feature calculations were conducted using MATLAB R2024a. A total of 37 fundamental features were extracted based on the existing literature. The raw Fp1–FP2 EEG channel data were utilized without additional preprocessing, such as filtering. However, to align with the practical constraints of portable EEG devices, which often have limited sampling capabilities, the original data—collected at 256 Hz—were down-sampled to 125 Hz. This step also reduces the computational complexity. The SS3 dataset was segmented into non-overlapping 30 s epochs, while the SS5 dataset was divided into non-overlapping 20 s epochs to comply with the sleep annotations provided by the experts. Then, the selected features were computed for each epoch.

#### 3.3.1. Time-Domain Features (3 Features)

Three time-domain features were extracted: skewness, kurtosis, and Higuchi Fractal Dimension (HFD). Skewness and kurtosis characterize the statistical distribution of the EEG signal, measuring asymmetry and peakedness, respectively. These features provide insight into transient signal behavior across sleep stages and were computed using standard statistical definitions.

HFD is a nonlinear measure of signal complexity, capturing the self-similarity and irregularity of the EEG time series. It was calculated following the original method proposed by Higuchi [42,43], which estimates fractal dimension from curve lengths over varying time scales. HFD has been shown to differentiate sleep stages based on temporal complexity in EEG signals.

#### 3.3.2. Hjorth Parameters (3 Features)

The Hjorth parameters—activity, mobility, and complexity—are widely used in EEG-based sleep analysis for their interpretability and low computational cost [22,25]. Activity represents the signal variance and provides a proxy for signal power, while mobility quantifies the dominant frequency content. Complexity measures the rate of change in frequency, helping to capture transitions between sleep stages. To enhance inter-subject comparability and reduce amplitude-related variability, the activity feature was standardized using z-score normalization across all epochs within each subject. This normalization was applied only to activity because it is directly influenced by the absolute amplitude of the EEG signal. In contrast, mobility and complexity are relative measures based on derivatives of the signal, making them inherently amplitude-invariant. All three parameters were computed using their standard time-domain definitions.

#### 3.3.3. Frequency-Domain Features (8 Features)

Spectral power ratios are widely used in sleep stage classification due to their ability to reflect frequency-specific EEG dynamics associated with distinct sleep stages [44,45,46]. The discrete Fourier transform (DFT) was applied to each epoch to analyze the signal in the frequency domain. The resulting power in the standard EEG frequency bands was computed, as shown in Table 2.

We acknowledge the overlap between the sigma (11–16 Hz) and alpha (8–13 Hz) bands, which could potentially introduce collinearity among frequency-domain features. However, this definition is consistent with the prior literature and established practice in sleep EEG analysis, where the sigma band is specifically chosen to encompass sleep spindle activity—primarily observed within the 12–15 Hz range during N2 sleep—distinct from the classical alpha rhythm (8–12 Hz) predominantly seen during wakefulness and REM sleep [13].

Based on the established literature, several PSD ratios were derived to capture the spectral characteristics associated with sleep stages. The computed ratios are shown in Table 3.

#### 3.3.4. Special Frequency-Domain Features (3 Features)

Inspired by prior studies [25,47], three additional spectral features were extracted to capture finer characteristics of EEG signals. Spectral Entropy quantifies the irregularity or complexity of the power spectrum, with higher values typically associated with wakefulness. Spectral Centroid (SC) represents the “center of mass” of the spectral distribution, indicating the average frequency around which the signal energy is concentrated. Higher SC values generally reflect the presence of higher-frequency components, as seen in REM sleep or in wake state. Spectral Roll-Off is the frequency below which a fixed proportion (95% was used in this study) of total spectral energy resides, helping distinguish low-frequency dominance (deep sleep) from higher-frequency dominance (wakefulness). All features were computed using standard definitions applied to normalized DFT-derived power spectra.

#### 3.3.5. Wavelet-Based Features (20 Features)

Wavelet components are crucial in sleep scoring as they provide a time–frequency representation of EEG signals, enabling the capture of transient features and frequency-specific patterns associated with different sleep stages [17,18,48]. Each epoch signal was decomposed using discrete wavelet transformation with the Daubechies-4 wavelet. The decomposition allowed the signal to be divided into five frequency bands, capturing both time- and frequency-domain characteristics. The selected bands of decomposed components and their importance are listed in Table 4.

Normalized wavelet energy and entropy were derived from each band’s power distribution to capture spectral compactness and irregularity. Hjorth mobility and complexity were calculated from the wavelet coefficients to describe the frequency content and its dynamic variation. These multi-scale features significantly enhance classification performance by encoding both spectral and temporal EEG characteristics across multiple frequency bands and time scales.

### 3.4. Transition-Based Feature Modeling Using a Markovian Framework

Inspired by the work carried out by Davies et al. [49], in this work, we introduce a transition-aware feature representation grounded in first-order Markov theory to capture the temporal dependencies between consecutive sleep stages. Unlike models that explicitly process sequences of epochs using recurrent architectures, our method encodes inter-epoch relationships via a transition vector derived from a subject-independent transition matrix. This approach allows us to incorporate probabilistic temporal structure in a computationally efficient, feedforward framework.

#### 3.4.1. Transition Matrix Construction

We begin by estimating a first-order transition matrix $T\in R^{K\times K}$ from the training data, where *K* is the number of sleep stage classes. Let $C=\{c_{1},c_{2},\ldots ,c_{K}\}$ denote the set of all sleep stages. Each entry $T_{ij}$ of the matrix represents the conditional probability of transitioning from stage $c_{i}$ to stage $c_{j}$ between consecutive epochs. Given the training sequence of sleep stage labels, the transition frequency from $c_{i}$ to $c_{j}$ is first counted, and then each row is normalized to form the conditional transition probabilities:

$$T_{ij}=P\left(S_{t}=c_{j}|S_{t-1}=c_{i}\right)=\frac{\mathrm{Count}(S_{t-1}=c_{i},S_{t}=c_{j})}{\sum_{j=1}^{K}\mathrm{Count}\left(S_{t-1}=c_{i},S_{t}=c_{j}\right)}.$$

This transition matrix captures the Markovian dynamics of sleep stage progression, reflecting expected temporal patterns such as frequent transitions between adjacent stages (e.g., N2 to N3) and rare transitions between non-adjacent stages (e.g., wake to N3).

#### 3.4.2. Transition Vector Feature

To incorporate this temporal information during classification, we compute a transition vector $v_{t}\in R^{K}$ for each epoch *t*. This vector is obtained by multiplying the softmax probability distribution from the previous epoch $p_{t-1}$ with the transition matrix T:

(4) $$v_{t}=p_{t-1}\cdot T.$$

Here, $p_{t-1}$ represents the model’s predicted probability distribution over all sleep stages at time t−1. For the first epoch, we initialize $p_{0}$ as a one-hot vector corresponding to the “wake” stage, based on the assumption that recordings typically begin during wakefulness.

The resulting transition vector $v_{t}$ serves as an additional feature input to our feedforward neural network. It provides a data-driven, probabilistic estimate of the likely sleep stage at epoch *t*, conditioned on the prior stage and learned transition dynamics, without the need for explicitly modeling long sequences.

#### 3.4.3. Integration with the Classification Framework

During Leave-One-Subject-Out (LOSO) cross-validation, a unique transition matrix T is computed using only the training subjects. The transition vector $v_{t}$ is then calculated for each epoch in the test subject using the model’s predicted softmax output from the previous epoch. This ensures that the test data remains fully isolated from the training data and prevents any leakage of ground truth labels.

**Figure 3:**
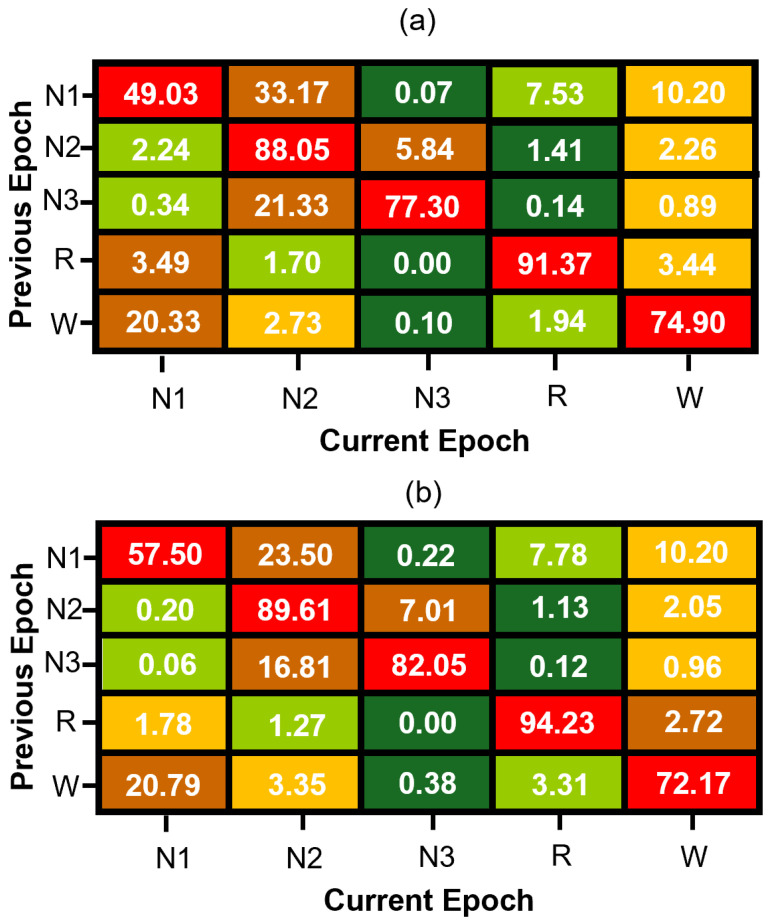
Normalized sleep stage transition matrices for (**a**) MASS SS3 and (**b**) MASS SS5 datasets. Each cell represents the percentage probability of transitioning to a current sleep stage (column) given the previous stage (row), computed from manually scored expert hypnograms. Rows are color-coded from green (lowest probability) to red (highest probability) to visually emphasize dominant and infrequent transitions within each sleep stage.

The transition vector is concatenated with conventional EEG-based features and used to train a “memory model” variant of our neural network. This approach enables the model to incorporate sequential context efficiently while preserving the simplicity and low computational cost of feedforward architectures. Figure 3 presents the estimated transition matrices for the MASS SS3 and SS5 datasets, which are in good agreement for the stage transitions with higher probability. The observed differences between these matrices can be primarily attributed to methodological differences between datasets. Specifically, the SS5 dataset was originally annotated using the six-stage R&K scoring system, and the subsequent merging of N3 and N4 stages into a single N3 class alters transition probabilities. Additionally, statistical variability—particularly pronounced in transitions with low probabilities due to the much smaller number of samples in their estimate when compared to the estimate of transition with much higher probability—further contributes to numerical discrepancies between the SS3 and SS5 matrices. Thus, the differences in these matrices reflect both methodological factors and statistical estimation errors rather than intrinsic physiological variability.

### 3.5. FNN Model

We implemented a subject-wise FNN classification framework using Python 3.10 and TensorFlow 2.19.0. All input features were standardized using StandardScaler, and sleep stage labels were encoded into integers using LabelEncoder, both from scikit-learn 1.6.1. The models were trained in a LOSO cross-validation manner, where a unique subject was used as the test set and the remaining subjects were used for training. To investigate the contribution of temporal information in sleep stage classification, we developed two separate FNN models: a no-memory model that relies solely on current-epoch EEG features and a memory-augmented model that incorporates temporal context via a transition vector. These models were trained independently using the same cross-validation framework and were later integrated during inference using a confidence-based decision strategy, as detailed in the following subsections.

#### 3.5.1. No-Memory Model

The baseline FNN model was trained using only EEG-derived features without any temporal context. The network architecture consisted of a fully connected structure with the following layers:

- Input layer: A dense layer with dimensionality equal to the number of input features, followed by ReLU activation.
- Hidden layers: Three dense layers with 128, 64, and 32 neurons, respectively, each using ReLU activation.
- Output layer: A softmax layer with *K* units corresponding to the number of sleep stages (K=5).

The model was compiled using the Adam optimizer with a learning rate of 0.001 and trained using sparse categorical cross-entropy loss. Early stopping was employed with a patience of 10 epochs to prevent overfitting. Feature vectors were independently normalized across subjects to ensure generalizability.

#### 3.5.2. Memory-Augmented Model with Transition Vector

**Figure 4:**
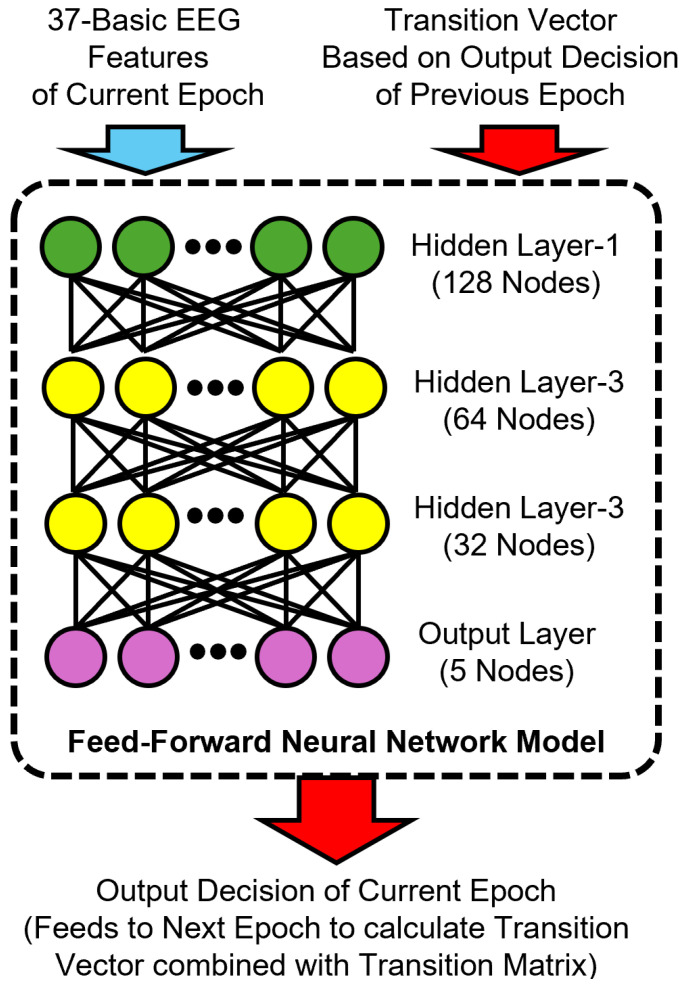
Architecture of the proposed memory-augmented feedforward neural network model for sleep stage classification. The input combines 37 basic EEG features of the current epoch with a transition vector derived from the previous epoch’s predicted output and a transition matrix. The model consists of three hidden layers with 128, 64, and 32 nodes, respectively, and a softmax output layer that predicts the current sleep stage. This prediction is then used to compute the transition vector for the next epoch.

To incorporate temporal information, we developed a memory-augmented variant of the FNN model by including a transition-based feature vector as input. Specifically, we computed a transition vector for each epoch based on the softmax output of the previous epoch and a dataset-level transition matrix T, as described in Section 3.5.1. Our memory-augmented FNN model architecture is shown in Figure 4.

Each transition vector $v_{t}=p_{t-1}\cdot T$ (where $p_{t-1}$ is the predicted distribution of the previous epoch) was concatenated to the EEG features to form the final input to the memory model. For the first epoch of each subject, the “wake” stage was assumed as the prior stage for computing the initial transition vector. This transition-aware representation was appended with one-hot-encoded previous epoch labels during training, but used only predicted softmax outputs during testing to simulate realistic deployment conditions. The memory-augmented FNN model retained the same architectural structure as the no-memory model but accepted a larger feature vector that included both conventional EEG features and the *K*-dimensional transition vector.

#### 3.5.3. Confidence-Guided Decision Strategy

During testing, both the no-memory and memory-augmented models independently generated sleep stage predictions for each epoch. For every prediction, the maximum softmax probability (i.e., model confidence) was recorded. A comprehensive analysis was performed to identify the optimal confidence threshold, evaluating the trade-off between classification coverage (proportion of classified epochs) and classification reliability (accuracy and Kappa values). Based on this threshold-level sweep—which is detailed in the Section 4 —a confidence threshold of 0.7 was selected as the optimal balance between accuracy, reliability, and minimal rejection rate, which is lower than the number of ambiguous epochs that have stage transitions.

Our decision algorithm operates as follows:

- The model with the higher confidence is selected if its confidence exceeds the threshold.
- If neither model exceeds the threshold, the epoch is marked as not classified (NC)

This inference-time integration of both models—selecting the prediction from the model with higher confidence when available—is referred to as the Combined Model throughout the remainder of this paper. This confidence-guided selection scheme enables fallback to the more reliable model for each epoch and avoids committing to low-confidence predictions, thereby improving both classification accuracy and reliability [50,51].

The classification NC is justified by the fact that a significant number of epochs consist of transitions between two different stages, which exhibit characteristics of both sleep stages.

### 3.6. Model Evaluation

Model performance was assessed using LOSO cross-validation across all available subjects. In each fold, one subject was used for testing, while the remaining subjects formed the training set. This subject-independent setup promotes robust generalization and simulates real-world deployment. Final predictions were generated using the confidence-based selection strategy described in Section 3.5.3. Epochs where both models yielded low-confidence outputs were marked as *NC* and excluded from metric computation.

Evaluation metrics included overall accuracy, Cohen’s kappa coefficient (κ), and class-wise F1 scores. Accuracy and kappa assessed overall model performance, while F1 scores were computed per class and macro-averaged to ensure balanced evaluation across sleep stages. Values of κ greater than 0.60 indicate substantial agreement, and values above 0.80 reflect near-perfect consistency [24,52,53].

## 4. Results

### 4.1. Autocovariance Functions and Autocorrelation Time

**Figure 5:**
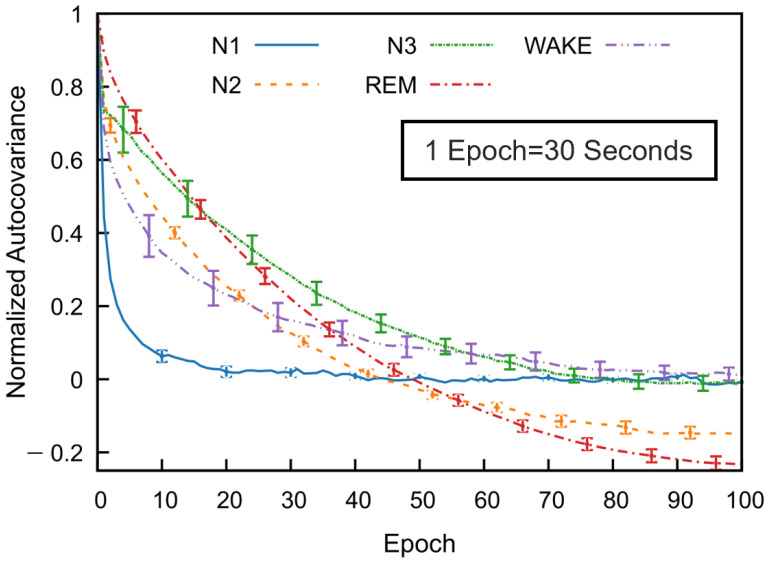
Normalized autocovariance (ACV) functions calculated for each sleep stage in the MASS SS3 dataset as a function of the epoch lag. For each subject and sleep stage, the binary ACV was computed and then averaged across 57 subjects. The resulting group-level mean ACV was normalized by its zero-lag value. Error bars represent the standard error of the mean and are shown at every 10-epoch lag.

To investigate temporal dependencies across sleep stages, we computed the normalized autocovariance function for each stage using data from multiple subjects. Figure 5 illustrates mean covariance functions across 53 subjects in the SS3 dataset, showing how sleep stage correlations decrease with the epoch lag. The error bars indicate the standard error of the mean, calculated at every tenth epoch.

All sleep stages exhibit a gradual decline in autocovariance with increasing epoch lag, indicating diminishing correlation with past stages as time progresses. Among the stages, REM sleep shows the highest autocovariance values as a function of the epoch lag, followed by N3 and N2, while N1 sleep has the lowest values. The wake stage exhibits an intermediate decay rate. These results reflect stage-specific differences in temporal correlation. For N1, wake, and N3 stages, the autocovariance function gradually approaches values near zero—fluctuating slightly above or below—at higher lags. In contrast, the autocovariance for N2 and REM decreases to negative values around the 50th epoch and remains consistently negative thereafter. Although a slight reduction in magnitude is observed, the function stabilizes and appears nearly constant beyond approximately 80 epochs. This trend was also consistently observed across subjects in the SS5 dataset.

**Figure 6:**
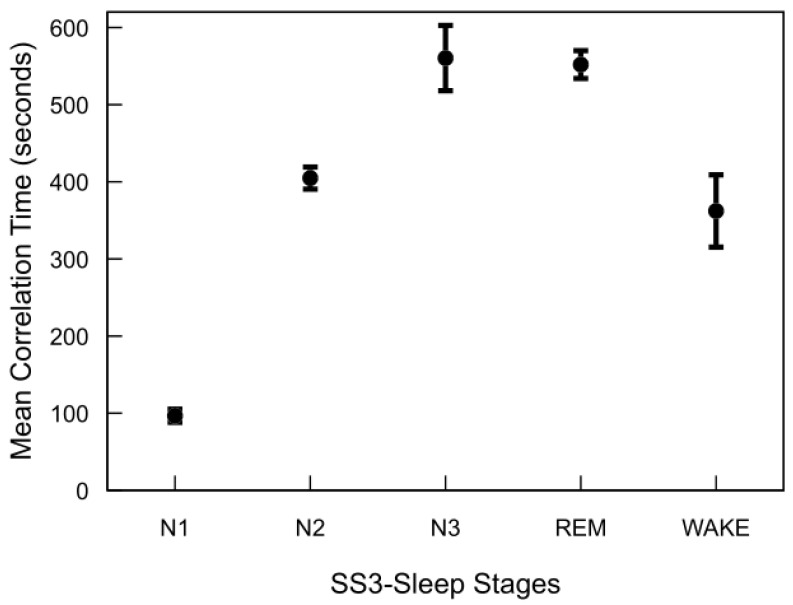
Autocorrelation time of sleep stages in the MASS SS3 dataset. For each subject and stage, autocorrelation time was computed by summing the normalized autocovariance function up to the first zero-crossing and multiplying by the 30 s epoch duration. The plot shows mean values across 57 subjects, with standard error bars.

Figure 6 illustrates the correlation time estimates for the MASS SS3 dataset, derived from the autocovariance functions of each sleep stage. REM, N2, and N3 stages exhibit significantly longer correlation times compared to N1 and wake. Although not shown, the MASS SS5 dataset demonstrates a similar trend. Across both datasets, correlation times for REM and deep sleep stages typically range from approximately 7 to 10 min, whereas N1 and wake stages are characterized by shorter correlation times of around 1 to 6 min. Notable inter-subject variability in correlation times was observed across all sleep stages. This variability was particularly pronounced in stages such as N3 and wake, reflecting underlying differences in EEG signal structure between individuals even within the same sleep stage.

The major finding of this analysis is that sleep stages—particularly REM and N3—exhibit significantly longer temporal dependencies compared to lighter stages (N1 and wake). This structured temporal correlation strongly supports integrating previous epoch information (memory) into classification models. By leveraging these inherent temporal patterns through feedback-based features, we can enhance sleep staging accuracy while maintaining low computational complexity, making such models highly suitable for practical, real-time medical applications like wearable sleep monitoring.

### 4.2. FNN Model Performance

**Figure 7:**
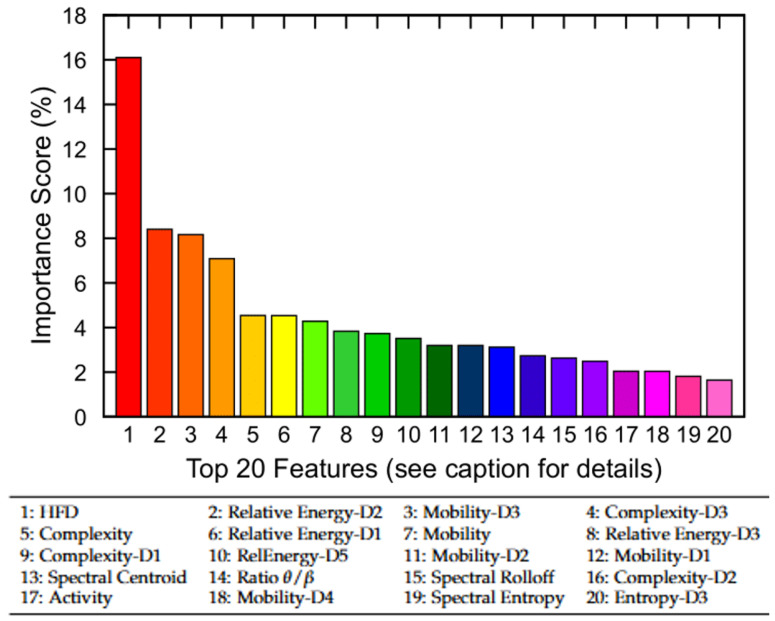
Permutation-based feature importance analysis using the no-memory FNN model. The top 20 EEG-derived features are ranked based on their relative contribution to model performance. Collectively, they account for 89% of total importance, while the remaining 17 features contribute 11%. Feature names correspond to nonlinear, time-domain, and frequency-domain metrics (see Section 3.3 for definitions).

To gain insight into the relative contribution of EEG-derived features, we first performed a permutation-based feature importance analysis using the trained no-memory FNN model. Figure 7 displays the top 20 features of the MASS SS3 dataset ranked by their importance scores, which collectively account for 89% of the total model contribution. The remaining 17 features contribute the remaining 11%. Notably, nonlinear complexity metrics such as HFD, Hjorth complexity across multiple scales (e.g., Complexity-D3), and relative wavelet-band power features (e.g., Relative Energy-D2 and Relative Energy-D1) emerged as the most informative. Although the current study utilizes all 37 EEG-derived features, these findings suggest that feature selection may offer computational benefits in resource-constrained settings. In particular, excluding low-importance features could reduce model complexity for mobile or embedded applications with only a marginal trade-off in classification performance.

**Figure 8:**
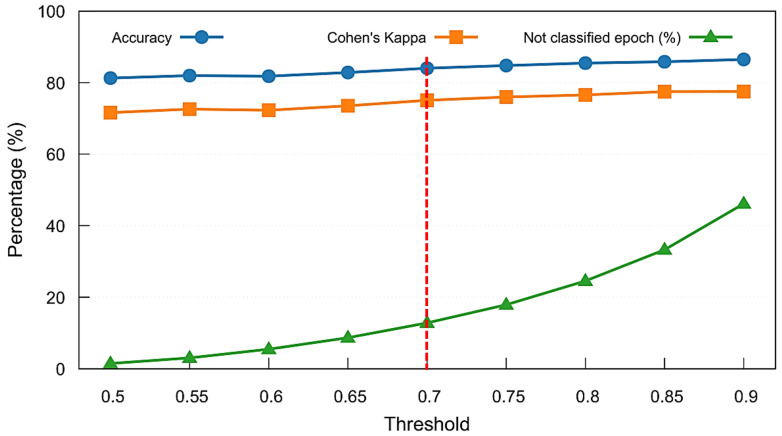
Threshold sweep analysis for the Combined Model on the MASS SS3 dataset. Mean accuracy, Cohen’s kappa, and percentage of unclassified (not classified) epochs are shown across varying softmax confidence thresholds. A threshold of 0.7 (marked with a red dashed line) was selected to maintain strong performance while limiting the rejection rate to approximately 13%.

Following the feature importance analysis, we evaluated the impact of confidence thresholding on classification outcomes using the Combined Model. Figure 8 presents the results of a threshold sweep on the MASS SS3 dataset, showing mean accuracy, Cohen’s kappa, and the proportion of unclassified (rejected) epochs across thresholds ranging from 0.5 to 0.9. As the confidence threshold increases, model reliability improves slightly, but the percentage of rejected epochs increases rapidly beyond 0.7, exceeding the number of ambiguous epochs that have stage transitions. To balance predictive performance with classification coverage, we selected a threshold of 0.7, marked by the vertical dashed red line, as a practical trade-off. This threshold was adopted throughout the remainder of our analyses, including model comparison and per-stage performance evaluation.

**Figure 9:**
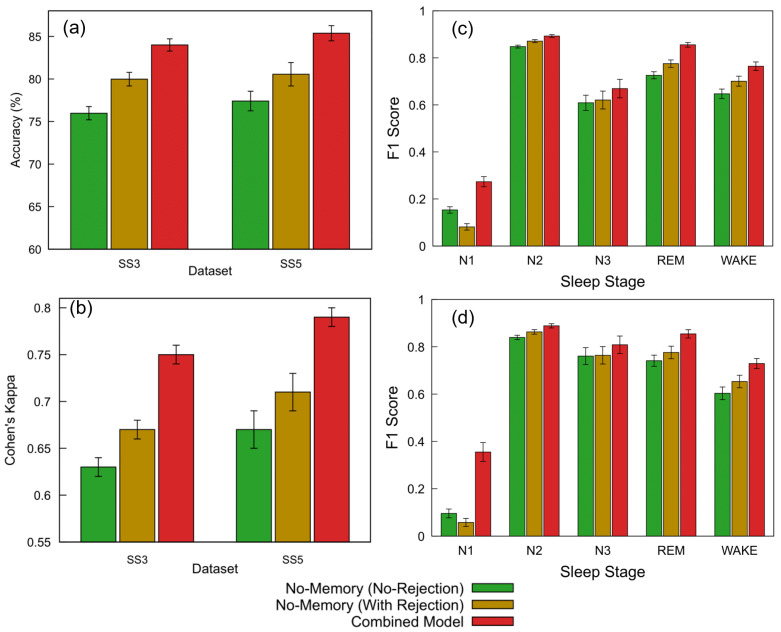
Performance comparison of FNN models across datasets and sleep staging metrics. (**a**) Accuracy and (**b**) Cohen’s kappa for the SS3 and SS5 datasets are shown for three configurations: no-memory (no rejection), no-memory (with rejection), and Combined Model. The no-memory (no rejection) model accepts all predictions. The Combined Model applies a 0.7 confidence threshold and selects the more confident output between the no-memory and memory-based models; predictions below this threshold are marked as NC and excluded from metric calculation (12.8% rejected in SS3, 9.4% in SS5). The no-memory (with rejection) model replicates the same rejection rate without memory fallback. (**c**,**d**) Show the per-class F1 scores across sleep stages (wake, N1, N2, N3, and REM) for SS3 and SS5, respectively. For each stage, bars represent performance for the three model configurations; error bars indicate standard error across subjects. The same rejection thresholds and confidence criteria as above were used for generating the Combined Model results.

Figure 9a,b and Table 5 summarize the classification performance of the proposed FNN models across the SS3 and SS5 datasets under three configurations: no-memory (no rejection), no-memory (with rejection), and Combined Model. Building on the threshold analysis, we applied the selected 0.7 confidence cutoff to each model’s softmax output to determine whether a prediction was accepted or rejected as unclassified (NC).

For the SS3 dataset, the no-memory model without any rejection initially achieved an accuracy of 75.98% and a Cohen’s kappa of 0.63, representing moderate agreement. We then introduced the Combined Model, which applies a confidence threshold of 0.7 and selects the more confident prediction between the no-memory and memory-augmented models. This approach resulted in 12.8% of the epochs being rejected due to low confidence and led to a substantial performance improvement—achieving an accuracy of 84.00% (+10.6%) and a kappa of 0.75 (+19.0%) relative to the baseline. To better understand the impact of rejection alone, we subsequently applied the same 12.8% rejection rate to the no-memory model without incorporating memory-based predictions. This improved its accuracy to 79.98% (+5.3%) and kappa to 0.67 (+6.3%). Standard errors for accuracy remained below 1% across all configurations, while kappa showed a consistent standard error of 0.01.

A similar evaluation sequence was followed for the SS5 dataset. The no-memory model without rejection initially achieved an accuracy of 77.41% and a Cohen’s kappa of 0.67. We then applied the Combined Model, which uses a confidence threshold of 0.7 to select the more confident prediction between the no-memory and memory-augmented models. This led to the rejection of 9.4% of the epochs and produced the highest performance, with an accuracy of 85.38% (+10.3%) and a kappa of 0.79 (+17.9%) relative to the baseline. To isolate the effect of rejection alone, we subsequently applied the same 9.4% rejection rate to the no-memory model without using memory-based predictions. This resulted in improved performance: 80.56% accuracy (+4.1%) and a kappa of 0.71 (+6.0%). Standard errors ranged from 0.89% to 1.38% for accuracy and from 0.01 to 0.02 for kappa.

These results clearly demonstrate that integrating temporal context via memory-based features substantially improves model accuracy and reliability compared to using instantaneous EEG features alone. Enhanced accuracy in identifying critical stages, such as REM and wake, significantly strengthens diagnostic confidence and supports personalized sleep disorder interventions. Further details on stage-specific performance are presented subsequently to illustrate the clinical applicability of the proposed hybrid model.

In addition to overall accuracy and agreement metrics, a stage-wise evaluation was conducted to assess model performance across individual sleep stages. Figure 9c,d present the F1 scores for the SS3 and SS5 datasets under three model configurations: no-memory (no rejection), no-memory (with rejection), and the Combined Model. Substantial improvements were observed in the REM and wake stages, which are particularly important for sleep monitoring applications. For REM, the F1 score increased from 0.73 to 0.86 in SS3 and from 0.74 to 0.85 in SS5 with the Combined Model. Similarly, the wake stage improved from 0.65 to 0.76 in SS3 and from 0.60 to 0.73 in SS5. While the N1 stage showed the largest relative gain, its absolute F1 score remained modest. The addition of a rejection strategy alone also provided moderate performance benefits. Error bars indicate the standard error across subjects.

The ability to reliably detect REM and wake stages is especially valuable in clinical sleep medicine, where accurate staging directly informs diagnosis and treatment planning for conditions such as REM sleep behavior disorder, insomnia, and sleep apnea. Improvements in F1 scores for these stages highlight the hybrid model’s practical advantage in real-world monitoring systems, particularly wearable devices where interpretability and robustness are critical. These results further demonstrate that confidence-based rejection and model combination can enhance per-stage reliability, especially for critical stages such as REM and wake.

**Figure 10:**
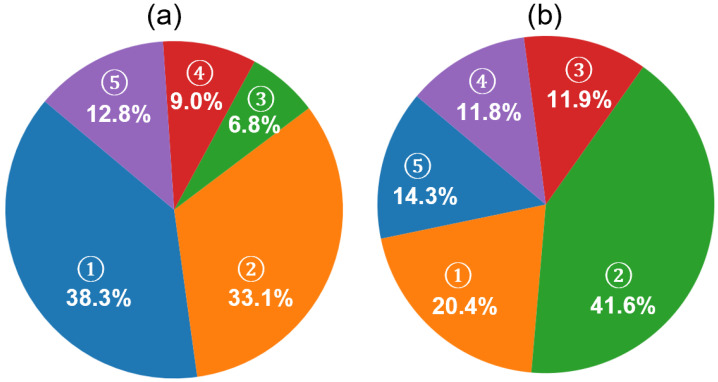
Analysis of prediction confidence and rejection composition in the MASS SS3 dataset using the proposed hybrid framework. (**a**) Contribution of model sources to final sleep stage predictions. Five categories are shown based on model confidence: (1) memory model dominant (both models confident; memory model has higher confidence, 38.3%), (2) no-memory model dominant (33.1%), (3) memory model only (6.8%), (4) no-memory model only (9.0%), and (5) not classified (both models below threshold, 12.8%). (**b**) Ground truth composition of unclassified (NC) epochs based on expert annotations. These NC epochs are those where neither model exceeded the confidence threshold. The relative distribution of expert-labeled stages within NC epochs includes (1) N1 (20.4%), (2) N2 (41.6%), (3) N3 (11.9%), (4) REM (11.8%), and (5) wake (14.3%).

While the improvements in F1 scores across individual sleep stages reflect the effectiveness of the hybrid classification framework, further insight into the model’s decision-making behavior is provided in Figure 10a. The pie chart illustrates the distribution of decision sources contributing to the final predictions. A significant portion of predictions (38.3%) were made when both models were confident, but the memory model exhibited higher certainty (memory model dominant), followed by 33.1% of predictions driven by the no-memory model under similar conditions (no-memory model dominant). In cases where only one model surpassed the confidence threshold, the system relied on that model alone, resulting in 6.8% (memory only) and 9.0% (no-memory only) of the total predictions. A relatively small fraction (12.8%) of epochs remained unclassified due to low confidence from both models.

This distribution highlights the complementary roles of the memory and no-memory models and supports the effectiveness of the confidence-based fallback strategy in enhancing prediction reliability while maintaining conservative decision boundaries. Such fallback logic can be especially useful in clinical settings, where minimizing misclassifications is often more critical than maximizing coverage. By rejecting uncertain predictions, the model enhances trustworthiness for downstream decision-making, such as staging-based sleep quality assessments or therapeutic interventions.

To further investigate the nature of the unclassified epochs described in Figure 10a, we examined their distribution based on expert-labeled ground truth, as shown in Figure 10b. Among these NC epochs—where neither model produced sufficiently confident predictions—the majority corresponded to N2 (41.6%) and N1 (20.4%) stages, followed by wake (14.4%), N3 (11.9%), and REM (11.8%). These results are consistent with the hypothesis that early-stage sleep (N1 and N2) and transitional periods contribute the most to classifier uncertainty, likely due to their intermediate signal features and low inter-rater agreement in manual scoring. The relatively lower representation of deep sleep and REM among the NC group also supports the effectiveness of the model in confidently detecting these more distinct stages.

In practical terms, this behavior indicates that our model is cautious during ambiguous or transitional sleep periods—an advantage in medical monitoring scenarios where incorrect staging may lead to misdiagnosis or ineffective treatment recommendations. Future work may focus on incorporating additional features or temporal smoothing to boost confidence during these uncertain periods.

## 5. Discussion

### 5.1. Autocovariance Functions and Autocorrelation Time

The observed differences in autocovariance and correlation time across sleep stages highlight the structured and cyclical nature of sleep architecture. The slow decay of the autocovariance function and the corresponding extended correlation times of the REM, N2, and N3 stages suggest a greater temporal continuity in these stages. This is consistent with the presence of organized neural oscillations, such as slow-wave activity during N3 sleep and theta rhythms during REM sleep, which are known to sustain over multiple epochs [3]. In contrast, the shorter correlation times in N1 and wakefulness reflect a more transient neural activity pattern, characterized by rapid fluctuations and decreased signal stability. N1, serving as a transitional stage between wakefulness and deeper sleep, naturally exhibits unstable dynamics, which aligns with its low autocovariance values [4]. The wake stage, influenced by environmental stimuli and voluntary movements, also shows reduced temporal consistency, supporting the observed short-memory characteristics.

Interestingly, the relatively constant autocovariance after reaching negative values in REM and N3 stages may indicate a quasi-stationary behavior in the transition patterns during these phases. The negative values in the autocovariance function suggest periodicity or anti-correlation at specific lags, reinforcing the idea that sleep is governed by underlying biological rhythms and cyclic transitions, particularly between REM and non-REM cycles [54]. Notably, the correlation times for N2 and REM stages were nearly identical across both SS3 and SS5 datasets, indicating consistent temporal structure in these stages regardless of dataset composition. However, direct comparison of the N3 stage is not feasible due to the inclusion of both N3 and N4 stages in SS5, whereas SS3 includes only N3. Additionally, a moderate deviation was observed in the correlation time of the wake stage, with SS3 exhibiting longer temporal persistence (∼5 min) compared to SS5 (∼3 min), which may reflect differences in the distribution or characteristics of wake epochs across the datasets. Furthermore, the variability in correlation time among subjects in stages like N3, wake, and N4 suggests individual differences in neural dynamics during sleep. These differences could be influenced by factors such as age, sleep quality, neurological conditions, or inter-individual variability in sleep regulation mechanisms [55,56].

Overall, these findings underscore the importance of modeling long-term temporal dependencies in sleep stage classification algorithms. Traditional models that treat sleep epochs as independent may overlook critical temporal patterns that are essential for accurate classification, especially for stages like REM and N3 that exhibit long autocorrelation times.

### 5.2. FNN Model Performance

#### 5.2.1. Interpretability and Limitations of Model Confidence and Predictions

The performance characteristics of the proposed FNN-based hybrid model can be better understood by analyzing its internal decision contributions and class-wise trends across datasets. As shown in Figure 10, the combined model leverages both memory-based and no-memory models for making final predictions based on softmax confidence levels. Notably, in 71.4% of the epochs, both models exceeded the confidence threshold, with the memory-based model contributing a dominant share of 38.3%. This indicates that the memory model—despite the risk of error propagation common in sequential architectures—remains a valuable component in capturing temporal patterns when supported by strong confidence. Furthermore, the hybrid architecture provides a safeguard against poor memory-based predictions by deferring to the no-memory model when necessary, thus balancing precision and robustness.

Figure 9c,d presents a comparison of F1 scores across sleep stages for different model configurations on the SS3 and SS5 datasets. As expected, the hybrid model consistently outperforms both standalone configurations, particularly in stages such as REM and wake. However, the N1 stage presents a unique case. While the overall F1 score for N1 improves when transitioning from the no-rejection to the hybrid model, we observe a drop in N1 performance when the same rejection threshold is applied to the no-memory model. This drop may be attributed to the inherently limited number of N1-labeled epochs in the datasets—only about 8% in SS3—and a disproportionately high rejection rate among these epochs. As shown in Figure 10b, 20.4% of the rejected epochs (NC) belong to the N1 class, suggesting that the model has lower confidence in this class likely due to its ambiguous characteristics and imbalanced representation. This observation underscores a common challenge in sleep staging: differentiating N1 from adjacent stages such as wake or N2, especially when data imbalance and transitional features are present [57].

The rejected (NC) epochs in our framework—12.8% in SS3 and 9.4% in SS5—represent segments where neither model achieved sufficient confidence (threshold = 0.7). These low-confidence epochs are likely enriched with transitional features between sleep stages. Notably, in the SS3 dataset, the average sleep stage transition rate is 17.7%, indicating that nearly one in six epochs lies at a boundary between two stages. Since sleep is analyzed using fixed 30 s epochs, transitions often occur within an epoch, not cleanly at its boundaries. This mixing of features from multiple stages within a single epoch could contribute to reduced model confidence. The lower rejection rate in the SS5 dataset (9.4%) may partially result from the shorter epoch duration (20 s), which increases the temporal resolution and reduces the likelihood of capturing mixed-stage signals within a single segment. This further supports the hypothesis that rejected epochs often reflect underlying physiological ambiguities rather than model failure.

#### 5.2.2. Benchmarking Against State-of-the-Art Models

The comparative performance metrics summarized in Table 6 provide a comprehensive benchmark of our proposed model against several state-of-the-art methods using various datasets, architectures, and EEG channel configurations. Despite achieving slightly lower performance compared to state-of-the-art models that leverage deep CNN and RNN architectures, our proposed FNN model demonstrates strong potential for deployment in computationally constrained, portable systems. For instance, while TinySleepNet and DeepSleepNet report overall accuracies of 87.5% and 86.2%, respectively, on the SS3 dataset, our model attains a competitive accuracy of 84.1% [23,58]. Similarly, the Cohen’s kappa score of our model (0.76) is marginally lower than the 0.82 and 0.80 reported by TinySleepNet and DeepSleepNet, respectively. This performance gap is acceptable and expected, considering that our FNN model is designed for efficiency and real-time inference on standalone mobile platforms where heavy CNN-RNN architectures may be computationally prohibitive.

A key factor contributing to the observed performance difference is the choice of EEG channel configuration. Most high-performing models utilize complex derivations such as F4-EOG (L) or C4-EOG (L), which provide stronger spatial resolution and benefit from the inclusion of eye movement signals [23,58,59,60]. In contrast, our model uses the Fp1-Fp2 channel, a challenging frontal derivation that is both closer to the eyes (thus prone to artifacts) and lacks the spatial depth of central or occipital leads. However, this channel is more practical and feasible for at-home, portable applications due to its easy placement and minimal user discomfort [62]. Notably, among the limited literature employing Fp1-Fp2 for automatic sleep staging, our model outperforms the decision tree approach by Popovic et al. (accuracy: 81.7%, kappa: 0.75) and even a CNN-RNN model by Leino et al., which reported only 79.7% accuracy and a kappa of 0.73 using a sequence length of 100 epochs [57,61]. This highlights the effectiveness of our model under challenging signal conditions.

Another distinguishing aspect of our approach is the minimal input sequence length used for prediction. Many existing models rely on long temporal contexts, with sequence lengths ranging from 10 to 100 epochs (equivalent to 5–50 min of EEG data). For example, IITNet and DeepSleepNet use 10- and 25-epoch input windows, respectively, while Leino et al.’s model requires 100 epochs [23,57,59]. These architectures, while improving temporal consistency, also introduce latency and dependency on extensive past data, making them less ideal for responsive systems. Our model, by contrast, processes and classifies each 30 s epoch using only the waveform acquired in that epoch (sequence length = 1), enabling immediate decision-making and simplifying memory requirements, which is advantageous for real-time mobile implementation. Despite this limitation, our model maintains competitive F1 scores across critical sleep stages, including REM (85.6%) and wake (76.5%), reinforcing its suitability for efficient and responsive sleep monitoring systems.

In addition to requiring minimal temporal input, the proposed model offers significant computational efficiency suitable for real-time applications. Implemented on a standard Intel Core i5-7200U CPU @2.5GHz without GPU support, the model achieved an average training time of 64.59 s per fold for the SS3 dataset and 32.97 s per fold for the SS5 dataset. The test was also efficient, requiring only 0.18 s per epoch for the SS3 dataset and 0.22 s per epoch for the SS5 dataset. Detailed information on the processing time of the proposed algorithm in these two datasets is provided in Table 7.

These times are notably lower than those reported by state-of-the-art models; for instance, IITNet required approximately 30 min per fold, and DeepSleepNet reported 3 h per node per fold using high-performance GPUs such as the NVIDIA RTX 2080 Ti or GTX 980 [23,59]. Furthermore, most prior studies do not report per-epoch testing times, making direct comparisons difficult. Nevertheless, these results emphasize the practicality of our model for deployment in portable and wearable systems, where computational resources and power consumption are limited.

To contextualize these results for practical deployment, we estimate the feasibility of implementing the model on resource-constrained microcontrollers commonly used in wearable devices. Modern low-power embedded systems such as the ARM Cortex-M7 or ESP32-S3 typically operate in the 240–600 MHz range [63,64]. Given our CPU-based testing time of 0.18–0.22 s per 30 s epoch, even modest optimization and quantization could reduce this latency to below 100 ms per epoch on embedded hardware using frameworks like TensorFlow Lite or Edge Impulse. This corresponds to an inference rate of approximately 10 epochs per second on a single-core microcontroller without hardware acceleration. Since our model processes pre-segmented 30 s EEG windows, this rate is more than sufficient to support real-time operation. The system can continuously buffer incoming EEG data and perform feature extraction and classification within the 30 s interval, ensuring timely and efficient decision-making.

## 6. Limitations

Despite demonstrating strong performance, our proposed hybrid classification framework has several limitations that must be acknowledged. First, the model was trained and evaluated exclusively on the MASS SS3 and SS5 datasets, which comprise healthy adult subjects recorded under standardized laboratory conditions. This limits the immediate generalizability of our findings to more heterogeneous or clinical populations, including individuals with sleep disorders, neurological comorbidities, or age-related changes in sleep architecture. Future work should validate the model on independent datasets and more diverse cohorts to assess its robustness in real-world settings. Additionally, while the use of the Fp1–Fp2 channel enhances practicality for wearable applications, it may exclude valuable spatial information from other brain regions relevant to pathological sleep patterns.

Second, the model continues to face challenges in accurately classifying the N1 stage—a well-known difficulty in sleep scoring due to its ambiguous EEG characteristics and low inter-rater agreement. Although our hybrid model and rejection strategy improve overall performance, the N1 stage remains the least confidently predicted, often contributing to rejected epochs. Addressing this issue may require integrating additional modalities (e.g., EOG or EMG), leveraging temporal smoothing strategies, or employing hierarchical classifiers that explicitly model transitional stages. Furthermore, while a full feature set was retained in this study, future implementations may explore reducing feature dimensionality for mobile deployment, accepting slight trade-offs in accuracy for gains in computational efficiency.

## 7. Conclusions

In this study, we investigated the temporal dependency of sleep stages through an autocorrelation analysis, confirming that prior sleep stages significantly influence subsequent classifications. To leverage this dependency, we introduced a lightweight hybrid framework for automatic sleep stage classification using EEG from the Fp1–Fp2 channel. By integrating a transition-aware temporal feature derived from the softmax output of the previous epoch and a learned transition matrix, we enabled a feedforward neural network to capture temporal dependencies without the complexity of sequential models. Our results demonstrated that this hybrid approach improved accuracy by over 10% and kappa by nearly 20% in both SS3 and SS5 datasets compared to baseline models. Substantial improvements were also observed in critical stages such as REM and wake, with F1 scores increasing by more than 10 points.

The confidence-based rejection strategy further enhanced prediction reliability, allowing the model to abstain from uncertain predictions—particularly in ambiguous transitional epochs—without compromising overall performance. The model’s efficient architecture, low computational overhead, and compatibility with minimally obtrusive EEG channels make it highly suitable for real-time implementation in wearable and portable devices. These characteristics support practical use in continuous home-based sleep tracking, early detection of sleep disorders, and resource-constrained clinical settings.

Future work will aim to improve generalizability to clinical populations, including those with sleep disorders, by validating the model on more diverse cohorts. Additionally, future efforts will focus on developing lightweight feature sets for wearable deployment and enhancing model confidence and accuracy during transitional stages such as N1 through adaptive learning strategies.

## Figures and Tables

**Table 1:** Summary of recent EEG-based sleep staging studies: model architectures, datasets, performance, and real-time suitability.

Study	Model Type	EEG Channels	Dataset	Performance Summary	Real Time?
Multi-Channel EEG Studies					
Sharma et al. [17]	Random Forest	C3-A2, C4-A1EMG, EOG	SHHS-1, SHHS-2	84.3–86.3% accuracy;effective but unsuitablefor wearable use.	No
Tzimourta et al. [18]	SVM, LDA	6 EEG	ISRUC	Max accuracy 75.29%;lacks temporal modeling;limited generalization.	No
Kong et al. [19]	CNN + RNN	Fpz-Cz, Pz-OzEMG, EOG	Sleep-EDF	80–82.7% accuracy;strong temporal modeling;high complexity.	No
Phan et al. [35]	Hierarchical RNN	EEG, EMG, EOG	MASS SS3	87.1% accuracy;excellent performance;high computational cost.	No
Single-Channel EEG Studies					
Zhao et al. [22]	ML (Various)	Single EEG	Various	Multiple classifiers;useful for simplified setups;lower accuracy range.	Partially
Supratak et al. [23]	CNN + Bi-LSTM	Single EEG	MASS, Sleep-EDF	82–86.2% accuracy;requires 25 epochs;not suitable for real time.	No
Liao et al. [33]	CNN	Fpz-Cz	Sleep-EDF	83.8% accuracy;lightweight design;suitable for mobile use.	Yes
Phan et al. [34]	Bi-RNN with attention	Fpz-Cz	Sleep-EDF	Up to 82.5% accuracy;good sequential modeling;high resource needs.	No
Huang et al. [38]	CNN + HMM	Fpz-Cz	Sleep-EDF	84.6% accuracy;HMM improves transitions;adds complexity.	Partially
Yang et al. [39]	CNN + CRF	Single EEG	Multiple datasets	Accuracy boost of 2.5–5.5%;context-aware refinement;increased inference time.	Partially
Proposed	FNN + Transition Vector	Fp1-Fp2	MASS SS3, SS5	84.1–85.4% accuracy;low complexity;ideal for real-time wearables.	Yes

Note: EEG = Electroencephalogram; EMG = Electromyogram; EOG = Electrooculogram; SHHS = Sleep Heart Health Study; ISRUC = Institute of Systems and Robotics—University of Coimbra; CNN = Convolutional Neural Network; RNN = Recurrent Neural Network; Bi-LSTM = Bidirectional Long Short-Term Memory; HMM = Hidden Markov Model; CRF = Conditional Random Field; FNN = Feedforward Neural Network.

**Table 2:** Frequency band definitions used in feature extraction.

Band	Range (Hz)	Band	Range (Hz)
Delta (δ)	0.5–4	Theta (θ)	4–8
Alpha (α)	8–13	Sigma (σ)	11–16
Beta (β)	13–30	Full band	0.5–30

**Table 3:** Spectral power ratios used as features.

Ratio No.	Ratio	Ratio No.	Ratio
1	δ/α	5	θ/β
2	δ/β	6	σ/δ
3	δ/θ	7	δ/(α+β)
4	θ/α	8	(δ+θ)/(α+β)

**Table 4:** Wavelet sub-bands from EEG decomposition.

Band	Frequency (Hz)	Importance
D1 (Beta/Gamma)	∼32–64	Low energy in REM, high in N1
D2 (Beta)	∼16–32	Helps distinguishing REM and N1
D3 (Alpha/Sigma)	∼8–16	Helps identify spindles in N2
D4 (Theta)	∼4–8	Higher presence in REM and N1
D5 (Delta)	∼0.5–4	Prominent in N2, reduced in REM

**Table 5:** Performance of FNN models on SS3 and SS5 datasets under rejection strategies.

Dataset	Metric	No-Mem (No Rej.)	No-Mem (Rej.)	Comb. Model (Rej.)
SS3	Accuracy (%)	75.98	79.98	84.00
	Std. Error	±0.78	±0.80	±0.72
	Kappa	0.63	0.67	0.75
	Std. Error	±0.01	±0.01	±0.01
SS5	Accuracy (%)	77.41	80.56	85.38
	Std. Error	±1.15	±1.38	±0.89
	Kappa	0.67	0.71	0.79
	Std. Error	±0.02	±0.02	±0.01

Note: Rejection thresholds: 12.8% for SS3 and 9.4% for SS5. Comb. Model applies confidence-based fallback to memory-based predictions. Standard error values are shown below each corresponding metric.

**Table 6:** Comparison of sleep stage classification performance across models and datasets. Accuracy, Cohen’s kappa, and per-class F1 scores are reported.

Method	Dataset	Model	Channel	Epoch	SL	Test	Overall Metrics		Per-Class F1 Score (F1)				
				(s)	(epoch)	Epochs	Accuracy	Kappa	W	N1	N2	N3	REM
IITNet [59]	SS3	CNN + RNN	F4-EOG (L)	30	10	57,395	86.6	0.80	86.1	54.4	91.3	86.0	86.2
DeepSleepNet [23]	SS3	CNN + RNN	F4-EOG (L)	30	25	58,600	86.2	0.80	87.3	59.8	90.3	81.5	89.3
TinySleepNet [58]	SS3	CNN + RNN	F4-EOG (L)	30	20	59,317	87.5	0.82	87.3	62.7	91.8	85.5	88.6
MixedNet [60]	SS3	MNN	F4-EOG (L)	30	5	59,066	85.9	–	84.6	56.3	90.7	84.8	86.1
TinySleepNet [58]	SS5	CNN + RNN	C4-EOG (L)	20	20	36,409	86.6	0.81	85.5	55.0	89.9	86.6	87.7
Popovic et al. [61]	Own	Decision Tree	Fp1-Fp2	30	1	15,934	81.7	0.75	74.82	47.0	86.9	87.9	86.3
Leino et al. [57]	Own	CNN + RNN	Fp1-Fp2	30	100	125,935	79.7	0.73	86.0	39.0	81.0	84.0	82.0
Proposed	SS3	FNN	Fp1-Fp2	30	1	54,581	84.1	0.76	76.5	27.4	89.3	72.1	85.6
Proposed	SS5	FNN	Fp1-Fp2	20	1	34,983	85.4	0.79	73.0	35.6	88.9	80.9	85.5

Note: This table presents a comparison of sleep stage classification performance across multiple models and datasets. CNN: Convolutional Neural Network; RNN: Recurrent Neural Network; FNN: Feedforward Neural Network; MNN: Mixed Neural Network, which combines Rectifier Neural Networks and RNNs. SL: Sequence Length, referring to the number of consecutive epochs used for a single prediction. A dash (–) indicates that the corresponding data was not reported in the original study. The reported metrics include overall accuracy, Cohen’s kappa (Kappa), and per-class F1 scores for wake (W), N1, N2, N3, and REM stages.

**Table 7:** Training and testing time statistics for SS3 and SS5 datasets.

Dataset	Training	Testing
SS3	Folds: 58 Total: 1.022 h Per fold: 64.59 s	Avg epochs: 956 Total: 2.776 h Per epoch: 0.18 s
SS5	Folds: 25 Total: 0.279 h Per fold: 32.97 s	Avg epochs: 1400 Total: 1.807 h Per epoch: 0.22 s

## Data Availability

The data presented in this study are available in the datasets SS3 and SS5 of the Montreal Archive of Sleep Studies (MASS). These data were derived from the following resources available in the public domain: https://borealisdata.ca (accessed on 30 January 2025).

## Abbreviations

The following abbreviations are used in this manuscript:

REM	Rapid Eye Movement
NREM	Non-Rapid Eye Movement
PSG	Polysomnography
EEG	Electroencephalography
EOG	Electrooculography
EMG	Electromyography
AASM	American Academy of Sleep Medicine
CNN	Convolutional Neural Network
RNNs	Recurrent Neural Networks
FNN	Feedforward Neural Network
MASS	Montreal Archive of Sleep Studies
R&K	Rechtschaffen and Kales
HFD	Higuchi Fractal Dimension
DFT	Discrete Fourier transform
PSD	Power Spectral Density
SE	Spectral Entropy
SC	Spectral Centroid
LOSO	Leave One Subject Out
NC	Not Classified
